# Availability of Essential Medicines in a Country in Conflict: A Quantitative Insight from Yemen

**DOI:** 10.3390/ijerph18010175

**Published:** 2020-12-29

**Authors:** Mohamed Izham Mohamed Ibrahim, Mohammed Alshakka, Nazeh Al-abd, Awsan Bahattab, Wafa Badulla

**Affiliations:** 1 Department of Clinical Pharmacy & Practice, Head of Research and Graduate Studies–Pharmacy, QU Health, Qatar University, Doha P.O. Box 2713, Qatar; 2 Section of Clinical Pharmacy, Department of Pharmaceutics, Faculty of Pharmacy, Aden University, Aden, Yemen; alshakka400@gmail.com; 3 Department of Para-Clinic, Faculty of Medicine and Health Science, University of Aden, Aden, Yemen; Nazehali78@gmail.com; 4 Department of Community Medicine and Public Health, Faculty of Medicine and Health Science, University of Aden, Aden, Yemen; osanhattab@gmail.com; 5 Department of Analytical Chemistry, Faculty of Pharmacy, University of Aden, Aden, Yemen; Aden.wf.77@gmail.com

**Keywords:** availability, accessibility, conflict, essential medicines, vital medicines, low- and middle-income countries, Yemen

## Abstract

Background: Medicine and medical supplies are often in short supply in countries suffering from the scourge of conflict. Effective medicine supply policies are lacking in many low- and middle-income countries (LMICs), particularly during conflict. This study aimed to assess the availability of essential medicines in both the public and private healthcare sectors. Methods: The study was conducted by administering a survey from November 2017 to February 2018 using the World Health Organization/Health Action International (WHO/HAI) guidelines and methodology. Thirty healthcare facilities in thirteen districts from three governorates in Yemen were included in the assessment of thirty essential medicines. The results were reported as frequencies and percentages of outlets with available medicines on the day of data collection. Results: A set of 30 vital and essential medicines were selected from the list of essential medicines that are used in healthcare centers in Yemen to treat prevalent diseases. In general, only 52.8% of the selected medicines were available in public and private healthcare settings. The distribution and availability of medicines in the three governorates were approximately equal. The availability of medicines was better in the private healthcare settings, specifically 73.3% in private hospitals and approximately 79.7% in private pharmacies. Conclusions: The availability of essential medicines during this state of conflict in three governorates in Yemen is low, in both public and private hospitals and healthcare centers. Many of the medications that were not available are used to treat chronic illnesses.

## 1. Introduction

The lack of adequate access to medicines in low- and middle-income countries (LMICs) is a critical problem. It has been reported than more than two billion individuals lack access to medicines [1]. Access to medicines is a basic human right. When people are prevented from obtaining medicines for their illnesses, particularly essential medicines, human rights are violated. The World Health Organization (WHO) has proposed the development and application of a national pharmaceutical policy to ensure accessibility to essential medicines. This policy is “a commitment to a goal and a guide of action” for pharmaceutical providers in the public and private sectors. Its intention is to ensure accessibility to essential medicines with proven quality and their proper use by the population, by addressing all aspects related to pharmaceuticals [2]. 

Yemen is one of the poorest countries in the Arab world, with a population of 28.5 million [3]. The United Nations Development Programme (UNDP) ranked Yemen 154th on the Human Development Index [4]. The situation became worse due to the Arab Spring in 2013 (i.e., which caused in regime changes in some Middle-Eastern countries), civil conflict, and the subsequent involvement of a Saudi-led multinational coalition [5]. Yemen had a weak healthcare system before the conflict, but this conflict exacerbated the situation in a short period and led to disaster throughout the healthcare system [6]. Yemen has suffered from the economic imbalances and instable government institutions for several years now, and thus it is currently facing substantial financial and economic difficulties, particularly during the present conflict [7]. 

Healthcare facilities in Yemen have been substantially affected [8]. Medication and equipment were destroyed. Shortages of medicines and skyrocketing prices have been reported [9]. Access to medicines is a problem that is important to millions of people. People are unable to obtain basic treatment and medicines for chronic illnesses such as cardiovascular illness, hypertension, and diabetes, or medicines for recurrent outbreaks of diseases such as cholera and dengue fever, resulting in high mortality and morbidity rates [10]. The WHO has recommended a few strategies to improve access to medicines, including procurement and supply-chain management, fair pricing, and management of intellectual property [11]. Availability is among the main factors used to measure individuals’ access to medicines for their illnesses. The WHO and Health Action International (HAI) have developed a standardized approach that has been used in several countries, including in Asia (e.g., China, India, Malaysia, Pakistan, Sri Lanka, Thailand, Yemen), Africa (e.g., Ethiopia, Malawi), North America (e.g., Haiti), etc. These studies focused on availability, prices, and affordability of essential medicines during normal conditions in the countries [12,13,14,15,16,17,18,19]. In the study in Yemen, a group of 30 medicines with pre-set dosage forms, strengths, and pack sizes that are relevant to the global burden of disease were used. Moreover, medicines were selected based on national importance. The survey was conducted in four cities, 20 public sector outlets, and 20 private pharmacies. The study indicated poor access to essential medicines, especially in the public sector.

The main purpose of this survey was to analyze the healthcare sector by specifically evaluating the availability of essential medicines during this state of conflict in selected cities, districts, and healthcare facilities in Yemen. To the best of our knowledge, research examining the availability and accessibility of medicines in Yemen during this difficult period is lacking. Therefore, the current study was conducted to recommend appropriate strategies to overcome these situations in the near future. 

## 2. Materials and Methods

### 2.1. Study Design

This study adopted and adapted the standardized approach developed by the WHO and HAI. For this study, availability was defined as follows: “the availability of medicines is reported as the percentage of medicine outlets in which the medicine was found on the day of data collection” [20,21]. The methodology allows for the measurement of important medicines’ (core and supplementary; lowest-priced generic and originator brand) availability in a reliable and standardized way. All data on the availability of medicines are collected in both the public (e.g., government hospitals, primary healthcare centers) and private sectors (e.g., licensed pharmacies) in different regions, states, or provinces of a country.

The present study was conducted from November 2017 to February 2018. The survey was performed in three cities of Yemen: Aden, Lahij, and Abyan. According to standard WHO/HAI methodology, six regions must be selected as “survey areas’’ (e.g., provinces, divisions, cities, districts, or counties) with estimated population coverage of about 0.1 to 0.25 million. All survey areas (e.g., three governorates in our case) must be reachable in a day from the main urban center (Aden in our case). However, we used a variant of the standard methodology because there was no conflict in these regions, while there was instability or inaccessibility in other areas due to the civil conflict. Yemen (with a population of approximately 29 million people) is a desert country in the Middle East, located on the southern tip of the Arabian Peninsula, bordered to the west by the Red Sea and the Bab-el-Mandeb Strait, in the north by Saudi Arabia, and in the northeast by Oman [22,23]. Aden is located along the north coast of the Gulf of Aden and lies on a peninsula enclosing the eastern side of Al-Tawāhī Harbour. It has a population of 1.8 million people. Lahij is a governorate of Yemen with a population of approximately 1 million people. This area is located between Aden and Ta′izz. The third study area was Abyan, which is a governorate in Yemen with a population of approximately half a million people. Each governorate is subdivided into a number of districts. For feasibility and convenience, several districts were selected for this study: Al-Mansoura, Al-Midan, Al-Katia, Al-Shaik, Khormakser, and Shaik Othman in Aden; Al-Hutah, Tuban, and Yawala in Lahij; and Al-Rumela Jaar, Khanfar, and Zengbar in Abyan. Four types of facility were surveyed: public hospitals (n = 2), private hospitals (n = 3), private pharmacies (n = 13), and public healthcare centers (n = 12). The main public hospital in the area was selected, and then four other public outlets from a list of all accessible public healthcare facilities were conveniently selected. The hospitals were mainly centralized in cities, while the private pharmacies and public healthcare centers were scattered throughout the country.

The set of medicines assessed was derived from the WHO model list of essential medicines [24]. According to WHO/HAI methodology, the selection of medicine for the surveys should include 30 core and supplementary medicines selected for each country based on local needs and disease burden. Medicines with specific formulation, dosage forms, and procured via donations were not included in the study, taking into account their infrequent usage for specific diseases and procedures, non-inclusion in the national essential medicines list (NEML), and difficulty in price estimation of donated medicines. Further, the set was compared with the Yemen list of essential medicines. Therefore, most of the medicines were chosen from Yemen NEML, considering local disease burden. A discussion was held, and the final set was approved by the research team from Aden University, composed of a general practitioner and two consultant physicians, based on epidemiological data available at the national level and in the study locations. The medicines were classified as V (vital), E (essential), or N (non-essential). VEN analysis of medicines is based on the criticality and utility for the patients in a country. Vital medicines are medicines that safe lives and prevent death or disability of patients. Essential medicines are non-vital, but important for significant illness that is less severe. Non-essential medicines are used for minor illnesses, with low therapeutic benefit but still important for patients. Patients will not die or experience a distressing condition without these medicines. 

The data collection procedure was designed, tested, and approved by the research team. We adapted the form and procedure of the WHO/HAI methodology. We used a variant of WHO/HAI methodology. We conducted the study only in three governorates instead of countrywide, in different parts of the country and not in the capital city, i.e., Sanaa. The study was also done at one point in time, as monitoring over a period of time was not feasible. In addition, we did not include other sectors such as non-governmental organizations. Three pharmacists who were trained as data collectors by two experienced faculty members (one of whom is an expert in pharmaceutical policy and medical supply management) were assigned to this study—one in each governorate. They were trained on how to communicate with the respective officers in charge in the facilities, what kind of data to collect, and how to measure the availability of the medicines. They visited the retail medicine outlets and recorded the availability of the selected medicines. The availability of each medicine was marked after physical checking (i.e., the availability of the medicines on the shelves) and proper documentation.

### 2.2. Ethical Considerations

The study procedure was approved by the Ethics Research Committee of the Faculty of Medicine and Health Sciences of Aden University (Ref: REC-42-2018), and permission was obtained from each of the healthcare organizations.

### 2.3. Data Analysis

The data were collected using the Excel program (Microsoft Corporation. (2018). Microsoft Excel. Retrieved from https://office.microsoft.com/excel) and analyzed using the SPSS^®^ software version 21.0 (IBM Corp. Released 2012. IBM SPSS Statistics for Windows, Version 21.0. Armonk, NY: IBM Corp.). Data collected and input by the data collectors were checked by the research team. Descriptive statistics, i.e., frequencies and percentages, were calculated to analyze the data. Quartiles were used to decide on the cut-off points of number of health facilities with medicines, i.e., 25%, which indicated a minimum acceptable number of healthcare facilities with medicine availability. Chi-square tests were carried out to determine the association between the medicines’ availability and cities and facilities. The alpha level was set at 0.05.

## 3. Results

All facilities in the three governorates that were selected in the study agreed to participate, as shown in Table 1. Thirty medicines from the Yemen list of essential medicines were selected in order to study their availability in public and private healthcare centers in three governorates in Yemen. The list of selected medicines is presented in Table 2. Antibacterials and cardiovascular-related medications were included among the thirty medicines. Fifteen of the medicines were vital, and the other half were classified as essential. All medicines were oral medications; less than 20% were administered in the form of either a syrup or suspension.

Table 3 summarizes the data for the availability of these medicines in 30 healthcare facilities in Yemen. The result indicated an average availability of these medicines per health facility of only 52.8%. Using quartiles, a cut-off point of 25% of facilities (n = 7.8), twenty-six (87%) of the medicines were not available at the time of the study in 25% or more of the facilities (eight or more facilities). Thirteen of the medicines (50% of 26) were vital medicines. Many of these medications are used to treat chronic illnesses.

The distribution of the availability among the three governorates was 59.6% in the 9 healthcare facilities in Aden, 52.1% in the 11 healthcare facilities in Lahij, and 48% in the 10 healthcare facilities in Abyan, as presented in Table 4. Among the three areas, Abyan was the most substantially affected. If a cut-off point of 25% of facilities in Abyan was applied, the majority of the medicines were not available at the time of study, with the exception of paracetamol, albendazole, amoxicillin, and metronidazole. However, in Lahij, the two least problematic medicines were paracetamol and amoxicillin, while many of the available medicines were not available in Aden, with the exception of ibuprofen, paracetamol, ciprofloxacin, metronidazole, ferrous sulfate, furosemide, ranitidine, oral dehydration salts, glibenclamide, and atorvastatin. In general, in all three areas, the non-available medicines were the medicines that are mainly used to treat chronic conditions. Further analysis was carried out to determine the association between the availability of medicines and the governorates. Significant associations were shown for two medicines, i.e., cloxacillin sodium (*p* = 0.048) and ferrous sulfate (*p* = 0.044). Low availability of cloxacillin sodium was found in the Aden and Lahij areas, while the availability of ferrous sulfate was low in the Lahij and Abyan areas.

The availability of the surveyed medicines in the public and private sectors is shown in Table 5. The availability of medicines in the public hospitals (n = 2 facilities) was generally 53.3%, while it was 73.3% in the private hospitals (n = 3 facilities). The private pharmacies had a 79.7% availability in the 13 facilities, while public healthcare centers had only 18.9% availability in 12 facilities. Both groups of public-sector facilities (hospitals and healthcare centers) were substantially affected. The medicines that were not available in the public hospitals at the time of the study were phenytoin sodium, praziquantel, glyceryl trinitrate, hydrochlorothiazide, nifedipine, salbutamol, atorvastatin, simvastatin, and methyldopa. Meanwhile, a greater number of medications were unavailable in the public healthcare centers during the study period, including chlorpheniramine, phenytoin sodium, praziquantel, cloxacillin sodium, folic acid, acetylsalicylic acid, clopidrogrel, glyceryl trinitrate, propranolol, hydrochlorothiazide, nifedipine, chlorpromazine HCl, salbutamol, digoxin, and methydopa. Further analysis was done to determine the association between medicine availability and healthcare facilities in the governorates. Chi-square tests indicated significant associations between the availability of medicines and the facilities, except for a few medicines, i.e., paracetamol, phenytoin sodium, cloxacillin sodium, ferrous sulfate, and oral rehydration salts.

## 4. Discussion

Our study was performed to determine the availability of thirty essential medicines during the present state of conflict in the selected governorates (i.e., Aden, Lahij, and Abyan), thirteen districts, and thirty healthcare facilities (in both the public and private sectors) in Yemen. The three governorates have a total population of approximately 3.5 million people. The studied areas and facilities covered urban and non-urban populations, while the healthcare settings covered primary, secondary, and tertiary care facilities.

Of the 30 medicines studied in 30 health facilities, slightly more than half of the medicines (52.8% per health facility) were available to the public. The percentage of availability of medicines in Aden (59.6%) was slightly better than in Lahij (52.1%) and Abyan (48.0%). Overall, the availability of medicines in the private sector (73.3%) was much better than in the public sector (53.3%), because these sectors are supported by and receive supplies from the investors; these medicines were available in approximately half of the public hospitals and approximately three-quarters of the private hospitals. Regarding the primary care settings, the private pharmacies had approximately four-fifths of the medicines (79.7%) compared to less than one-fifth (18.9%) in the public health centers.

The effect of the ongoing conflict in Yemen on access to medicine has not been sufficiently assessed. The effects on health, social, and economic aspects are detrimental. Reports have clearly shown the devastating effects of conflict on basic healthcare infrastructure and services, which may include shortages of lifesaving medicines [25,26]. Healthcare personnel are struggling to deliver fundamental basic healthcare services. Yemen has a weak governance and healthcare system that has become more fragile during this conflict, when the country has to import 80–90% of its medicines [26]. The pharmaceutical infrastructure, already in a poor state, has deteriorated further during the conflict. This situation affects the access to medicines, particularly essential medicines. An analysis of the availability of medicines at various sites, i.e., in different geographical areas and healthcare settings, is important to provide information to the different authorities that will assist them in taking the appropriate actions. The entire healthcare system in Yemen is governed and monitored by the Ministry of Public Health and Population (MOPHP).

Essential medicines should be available to all populations for free or at an affordable price as a fundamental component of a good healthcare system [27]. Access is defined as the ability to obtain medicines that are regularly available at affordable prices from the public or private healthcare system [28]. Approximately a third of residents worldwide lack access to required medicines [29]. The situation is problematic in most LMICs, and even worse in countries in conflict. Yemen is currently suffering the consequences of the conflict in 2015. Access to essential medicines became almost impossible for most people because of the lack of medicines available in public healthcare centers and high prices in private pharmacies, particularly when compounded with the low standard of living and the lack of health insurance.

The aim of the current study was to highlight the problem of the unavailability of essential medicines and to distinguish the dimensions of the problem in order to develop appropriate strategies and policies to solve the problem in cooperation with the competent authorities.

The findings of this survey indicated low availability of vital and essential medicines in the surveyed healthcare centers in general, and specifically in the public healthcare sector. Approximately half of the medicines (52.8%) were available, which was lower than the WHO (80%) voluntary target for the availability of affordable essential medicines, including generics, used to treat major non-communicable diseases (NCDs) in the public and private sectors of countries by 2025 [30]. A shortage in the availability of some essential medicines for chronic diseases, such as allergy, epilepsy, hypertension, cardiac diseases, asthma, psychiatric illness, and hypercholesterolemia, was observed. These diseases are unlike acute diseases because they generally require continuous, lifelong treatment. Other surveys conducted using the WHO/HAI methodology in some LMICs, including Yemen in 2006, also identified shortages in the availability and affordability of medicines for chronic diseases [31,32,33,34,35], as well as limited supplies of some medicines used to treat infectious diseases. These types of medicines must be available and affordable in both public and private healthcare facilities, particularly in LMICs. The previous Yemeni study indicated that no innovator brands were found in the public sector, and the availability of generic medicines was very poor. Three-quarters of the medicines were found in less than 12% of the studied pharmacy outlets. In the same study, the researchers found that the availability of medicines in public pharmacies was poorer than in private pharmacies. The availability of generic medicines in the private sector was better than that of branded medicines [19]. Overall, the situation in the present study was not much difference from the past. This suboptimal availability of medicines is probably a major factor contributing to the high morbidity and mortality rates in the country.

Although the average availability of the medicines in the private sector, including hospitals (73.7%) and pharmacies (79.7%), was higher than in the public sector, the price and affordability should be considered. These aspects should be studied, as both price and affordability also affect access to medicines. The lack of affordable medicine in the public sector (e.g., medicine shortages) and slightly increased access to higher-priced medicine in the private sector are complementary issues. Patients will end up without medicines necessary to treat their illnesses. Fundamental improvements in the availability of essential medicines can be achieved if the underlying causes are identified, such as suboptimal availability in the public sector. Higher availability of essential medicines was identified in private hospitals and private pharmacies. These entities are business-oriented, profit-making organizations. Price is a concern. The high price of medicines in the private sector is another barrier. 

Several studies reported similar outcomes. Xi et al. evaluated the availability, affordability, and prices of essential medicines in Jiangsu Province, China after the implementation of the policy in 2009. In terms of availability, the essential medicines policy was effective in the primary healthcare settings, but not in the secondary and tertiary settings. A low availability of medicines was reported in these settings [15]. Anson et al. observed a lower availability of medicines in the Guatemalan public sector than in the private sector [36]. Robertson and coworkers conducted a review of studies performed in Tanzania and found that all the studies showed a suboptimal availability of medicines for non-communicable diseases [37]. According to a Brazilian study, the public sector suffered from low availability of essential medicines that failed to meet the constitutional requirement [38]. This is an issue in LMICs, and is likely more of an issue in conflict zones; the issue becomes particularly serious when a country has lack of commitment and effective policy.

Extensive investigations from a number of competent authorities (governmental, non-governmental, and international agencies) are required to initiate and design strategies that will ensure the availability of essential medicines to all people. Health policy should be completely developed and enforced in all sectors and levels. An appropriate supply of free or low-cost essential medicines in the public sector is one of the main determinants of medicine accessibility, and requires consideration and effort from the relevant authorities. Systems to determine the prices of medicines in the private sector and to reduce or eliminate taxes on the essential medicines must be established to ensure accessibility and affordability. In addition, an initiative to improve local manufacturing qualifications and abilities in the production of essential medicines to cover the needs of the people must be implemented, as stated by the WHO [39]. According to the Annual Report for Yemen SBDMA and the study by Alshakka et al., the local pharmaceutical industry in Yemen covers approximately 10–20% of the total market [40,41]; other medicines must be imported. The WHO program that supports the availability of medicines for some endemic diseases, such as HIV/AIDS and other diseases, has proven to be effective [42,43], and thus the expansion of this program to include the availability of essential medicines in poor countries and countries in conflict, such as Yemen, would be suitable. The healthcare needs of people in a state of conflict become increasingly complex to diagnose and manage [44]. The effects of a conflict on an individual’s health range from traumatic injuries to a loss of continuity of care for chronic illnesses. The approaches used by the WHO in conflict states such as Syria should be assessed, adopted, or adapted [45]. Moreover, the Geneva Convention IV and Additional Protocol I explain the provisions of humanitarian assistance, such as access to medicine [46]. In brief, the goals are to increase the proportion of available essential medicines to the people of Yemen, ensuring affordable medicines for patients and institutional change. 

The weaknesses in the pharmaceutical sector in Yemen and the needs of the same must be mapped; this assessment is important to allow strategizing and prioritization of areas of interventions designed to address the accessibility of medicines, particularly when due to a lack of financial resources [47]. The civilians in Yemen deserve a better healthcare system and access to quality essential medicines. The Yemeni government could consider the following courses of action: to prioritize the medicine budget and focus more on the essential medicines; to continuously update the essential medicines list; to procure and stock cost-effective generic medicines; and to show a higher preference for generic medicines rather than expensive originator-brand medicines.

The study was limited to three of the 21 governorates of Yemen; however, it provides an estimate of the poor situation of essential medicines’ availability in the country. The findings cannot be generalized to the whole of Yemen, given that the study only included three cities and had a small sample size (i.e., medicine outlets), and therefore only covered these three cities. Moreover, the probability selection method of study settings was not able to be applied. These problems were due to the conflict, which might have affected the safety of the researchers. The sample was not representative of the country. However, it is important to indicate that problems of medicine availability do exist. The study should be expanded to other cities and governorates when the safety problem is resolved. Second, the study only focused on 30 medicines, which are mostly administered in oral form. An expanded study should also consider other medicines and focus on certain diseases. It would also be useful to expand this study to investigate what lead to the current situation using regression or other estimation methods. Lastly, the study was not a pre–post design, i.e., before and after the conflict. Hence, it is difficult to provide an appropriate judgement of whether the insufficient supply of essential medicines, especially in the public sector, is the result of domestic conflict or failures of the medical system. Continuous observation and estimates of strategies and policy interventions are critical to improve access to essential medicines. 

## 5. Conclusions

In summary, the current study indicated a low availability of vital and essential medicines in 30 public and private healthcare facilities in three governorates in Yemen. The lack of availability of essential medicines was worse in the public sector than in the private sector. 

## Figures and Tables

**Table 1:** Cities, districts, and types of facility included in the study.

City (n = 3)	District (n = 13)	Type of Healthcare Facility				
		Public Hospital	Private Hospital	Private Pharmacy	Public Healthcare Center	Number of Facilities
Aden (popln = 1.8 m)	Al-Mansoura	-	1	1	1	3
	Al-Midan	-	-	1	1	2
	Al-Katia	-	-	-	1	1
	Al-Shaik	-	-	-	1	1
	Khormakser	-	-	1	-	1
	Shaik Othman	-	-	1	-	1
**Subtotal**						**9**
Lahij	Al-Hutah	1	-	5	-	6
	Tuban	-	1	-	3	4
(popln = 0.9 m)	Yawala	-	-	-	1	1
**Subtotal**						**11**
Abyan (popln = 0.5 m)	Al-Rumela	-	-	-	1	1
	Jaar	-	1	2	-	3
	Khanfar	1	-	-	3	4
	Zengbar	-	-	2	-	2
**Subtotal**						**10**
**Total**		**2**	**3**	**13**	**12**	**30**

**Table 2:** List of essential medicines included in the study.

No	Class of Medicines	Medicines	Formulation	Strength	VEN
1	Antibacterials	Amoxicillin	caps	500 mg	V
2	Psychotherapeutic	Chlorpromazine HCl	tab	25/100 mg	V
3	Antibacterials	Ciprofloxacin	tab	500 mg	V
4	Anticoagulation	Clopidrogrel	tab	5 mg	V
5	Antibacterials	Cloxacillin sodium	susp	250 mg/5 mL	V
6	Cardiac glycosides	Digoxin	tab	0.25 mg	V
7	Antibacterials	Erythromycin	susp	200 mg/5 mL	V
8	Diuretics	Furosemide	tab	40 mg	V
9	Antihypertensive/ Diuretic	Hydrochlorothiazide	tab	25/50 mg	V
10	Antidiabetic	Metformin	tab	500 mg	V
11	Antihypertensive	Methyldopa	tab	250 mg	V
12	Antimoebic	Metronidazole	tab	250/500 mg	V
13	Antidiarrhea	Oral dehydration salts	powder	NA	V
14	Anthelminthics	Praziquantel	tab	600 mg	V
15	Antiarrythmic/ Antihypertensive	Propanolol	tab	10/40 mg	V
16	Anticoagulation/ Antithrombolytic	Acetylsalicylic acid	tab	75/100 mg	E
17	Anthelminthics	Albendazole	tab	200/400 mg	E
18	Anticholesterol	Atorvastatin	tab	10/20 mg	E
19	Antiallergic	Chlorpheniramine	tab	4 mg	E
20	Antianaemic	Ferrous sulfate	tab	65/125 mg	E
21	Antianaemic	Folic acid	tab	5 mg	E
22	Antidiabetic	Glibenclamide	tab	5 mg	E
23	Antianginal	Glyceryl trinitrate	sub/tab	5/10 mg	E
24	Analgesic	Ibuprofen	tab	400 mg	E
25	Antihypertensive	Nifedipine	caps/tab	10/20 mg	E
26	Antipyretic	Paracetamol	syrup	125/250 mg/5 mL	E
27	Antiepileptics	Phenytoin sod	syrup	30 mg/5 mL	E
28	Antacid	Ranitidine	tab	150 mg	E
29	Antiasthmatic	Salbutamol	syrup	2 mg/5 mL	E
30	Anticholesterol	Simvastatin	tab	10/20 mg	E

Note: V = vital, E = essential, and N = non-essential.

**Table 3:** Availability of the essential medicines surveyed in this study.

			Availability of Medicines Per Healthcare Facility (n = 30) n (%)		
No	Class of Medicines	Medicines	Yes	No	VEN
1	Antibacterials	Amoxicillin	25 (83)	5 (17)	V
2	Psychotherapeutic	Chlorpromazine HCl	13 (43)	17 (57)	V
3	Antibacterials	Ciprofloxacin	22 (73)	8 (27)	V
4	Anticoagulation	Clopidrogrel	13 (43)	17 (57)	V
5	Antibacterials	Cloxacillin sodium	8 (27)	22 (73)	V
6	Cardiac glycosides	Digoxin	15 (50)	15 (50)	V
7	Antibacterials	Erythromycin	19 (63)	11 (37)	V
8	Diuretics	Furosemide	21 (70)	9 (30)	V
9	Antihypertensive/ Diuretic	Hydrochlorothiazide	7 (23)	23 (77)	V
10	Antidiabetic	Metformin	18 (60)	12 (40)	V
11	Antihypertensive	Methyldopa	16 (53)	14 (47)	V
12	Antimoebic	Metronidazole	25 (83)	5 (17)	V
13	Antidiarrhoea	Oral dehydration salts	18 (60)	12 (40)	V
14	Anthelminthics	Praziquantel	5 (17)	25 (83)	V
15	Antiarrythmic/ Antihypertensive	Propanolol	10 (33)	20 (67)	V
16	Anticoagulation/ Antithrombolytic	Acetylsalicylic acid	16 (53)	14 (47)	E
17	Anthelminthics	Albendazole	19 (63)	11 (37)	E
18	Anticholesterol	Atorvastatin	17 (57)	13 (43)	E
19	Antiallergic	Chlorpheniramine	15 (50)	15 (50)	E
20	Antianaemic	Ferrous sulfate	13 (43)	17 (57)	E
21	Antianaemic	Folic acid	16 (53)	14 (47)	E
22	Antidiabetic	Glibenclamide	22 (73)	8 (27)	E
23	Antianginal	Glyceryl trinitrate	6 (20)	24 (80)	E
24	Analgesic	Ibuprofen	24 (80)	6 (20)	E
25	Antihypertensive	Nifedipine	14 (47)	16 (53)	E
26	Antipyretic	Paracetamol	28 (93)	2 (7)	E
27	Antiepileptics	Phenytoin sod	2 (7)	28 (93)	E
28	Antacid	Ranitidine	22 (73)	8 (27)	E
29	Antiasthmatic	Salbutamol	14 (47)	16 (53)	E
30	Anticholesterol	Simvastatin	12 (40)	18 (60)	E
	**Total**		**475 (52.8%)**	**425 (47.2%)**	

Note: V = vital, E = essential, and N = non-essential.

**Table 4:** Availability of the essential medicines surveyed in the three cities.

		Aden (No of Facilities = 9)		Lahij (No of Facilities = 11)		Abyan (No of Facilities = 10)		*p*-Value
No	Medicines	Yes n (%)	No n (%)	Yes n (%)	No n (%)	Yes n (%)	No n (%)	
1	Ibuprofen	9 (100)	0 (0.0)	8 (72.7)	3 (27.3)	7 (70)	3 (30)	0.198
2	Paracetamol	9 (100)	0 (0.0)	10 (90.9)	1 (9.1)	9 (90)	1 (10)	0.630
3	Chlorpheniramine	4 (44.4)	5 (55.6)	7 (63.6)	4 (36.4)	4 (40)	6 (60)	0.514
4	Phenytoin sod	1 (11.1)	8 (88.9)	0 (0.0)	11 (100)	1 (10)	9 (90)	0.535
5	Praziquantel	2 (22.2)	7 (77.8)	1 (19.1)	10 (90.9)	2 (20)	8 (80)	0.693
6	Albendazole	4 (44.4)	5 (55.6)	7 (63.63)	4 (36.4)	8 (80)	2 (20)	0.275
7	Amoxicillin	6 (66.7)	3 (33.3)	9 (81.8)	2 (18.2)	10 (100)	0 (0.0)	0.148
8	Cloxacillin sodium	0 (0.0)	9 (100)	3 (27.3)	8 (72.7)	5 (50)	5 (50)	**0.048**
9	Erythromycin	4 (44.4)	5 (55.6)	8 (72.7)	3 (27.3)	7 (70)	3 (30)	0.369
10	Ciprofloxacin	9 (100)	0 (0.0)	7 (63.6)	4 (36.4)	6 (60)	4 (40)	0.095
11	Metronidazole	9 (100)	0 (0.0)	7 (63.6)	4 (36.4)	9 (90)	1 (10)	0.075
12	Ferrous sulfate	7 (77.8)	2 (22.2)	3 (27.3)	8 (72.7)	3 (30)	7 (70)	**0.044**
13	Folic acid	5 (55.6)	4 (44.4)	7 (63.6)	4 (36.4)	4 (40)	6 (60)	0.548
14	Acetylsalicylic acid	4 (44.4)	5 (55.6)	7 (63.6)	4 (36.4)	5 (50)	5 (50)	0.670
15	Clopidrogrel	3 (33.3)	6 (66.7)	6 (54.5)	5 (45.5)	4 (40)	6 (60)	0.614
16	Glyceryl trinitrate	2 (22.2)	7 (77.8)	2 (18.2)	9 (81.8)	2 (20)	8 (80)	0.975
17	Propanolol	4 (44.4)	5 (55.6)	5 (45.5)	6 (54.5)	1 (10)	9 (90)	0.159
18	Hydrochlorothiazide	2 (22.2)	7 (77.8)	3 (27.3)	8 (72.7)	2 (20)	8 (80)	0.921
19	Nifedipine	5 (55.6)	4 (44.4)	5 (45.5)	6 (54.5)	4 (40)	6 (60)	0.790
20	Furosemide	9 (100)	0 (0.0)	6 (54.5)	5 (45.5)	6 (60)	4 (40)	0.061
21	Ranitidine	9 (100)	0 (0.0)	7 (63.6)	4 (36.4)	6 (60)	4 (40)	0.095
22	Oral dehydration salts	8 (88.9)	1 (11.1)	6 (54.5)	5 (45.5)	4 (40)	6 (60)	0.085
23	Glibenclamide	9 (100)	0 (0.0)	7 (63.6)	4 (36.4)	6 (60)	4 (40)	0.095
24	Metformin	6 (66.7)	3 (33.3)	8 (72.7)	3 (27.3)	4 (40)	6 (60)	0.276
25	Chlorpromazine HCl	3 (33.3)	6 (66.7)	6 (54.5)	5 (45.5)	4 (40)	6 (60)	0.614
26	Salbutamol	5 (55.6)	4 (44.4)	6 (54.5)	5 (45.5)	5 (50)	5 (50)	0.966
27	Digoxin	5 (55.6)	4 (44.4)	5 (45.5)	6 (54.5)	5 (50)	5 (50)	0.904
28	Atorvastatin	7 (77.8)	2 (22.2)	6 (54.5)	5 (45.5)	4 (40)	6 (60)	0.248
29	Simvastatin	6 (66.7)	3 (33.3)	4 (36.4)	7 (63.6)	2 (20)	8 (80)	0.111
30	Methyldopa	5 (55.6)	4 (44.4)	6 (54.5)	5 (45.5)	5 (50)	5 (50)	0.966
	**Total**	**161 (59.6)**	**109 (40.4)**	**172 (52.1)**	**158 (47.9)**	**144 (48.0)**	**156 (52.0)**	

*p*-values in bold mean ‘significant’.

**Table 5:** Availability of essential medicines surveyed in the different health facilities.

		Public Hospital (No of Facilities = 2)		Private Hospital (No of Facilities = 3)		Private Pharmacy (No of Facilities = 13)		Public Health Center (No of Facilities = 12)		*p*-Value
No	Medicines	Yes	No	Yes	No	Yes	No	Yes	No	
1	Ibuprofen	2 (100%)	0 (0.0%)	3 (100%)	0 (0.0%)	13 (100%)	0 (0.0%)	6 (50%)	6 (50%)	**0.010**
2	Paracetamol	2 (100%)	0 (0.0%)	3 (100%)	0 (0.0%)	13 (100%)	0 (0.0%)	10 (83.3%)	2 (16.7%)	0.360
3	Chlorpheniramine	1 (50%)	1 (50%)	3 (100%)	0 (0.0%)	11 (84.6%)	2 (15.4%)	0 (0.0%)	12 (100%)	**0.000**
4	Phenytoin sod	0 (0.0%)	2 (100%)	0 (0.0%)	3 (100%)	2 (15.4%)	11 (84.6%)	0 (0.0%)	12 (100%)	0.423
5	Praziquantel	0 (0.0%)	2 (100%)	0 (0.0%)	3 (100%)	5 (38.5%)	8 (61.5%)	0 (0.0%)	12 (100%)	**0.049**
6	Albendazole	1 (50%)	1 (50%)	3 (100%)	0 (0.0%)	12 (92.3%)	1 (7.7%)	3 (25%)	9 (75%)	**0.003**
7	Amoxicillin	2 (100%)	0 (0.0%)	3 (100%)	0 (0.0%)	13 (100%)	0 (0.0%)	7 (58.3%)	5 (41.7%)	**0.029**
8	Cloxacillin sodium	1 (50%)	1 (50%)	1 (33.3%)	2 (66.7%)	6 (46.2%)	7 (53.8%)	0 (0.0%)	12 (100%)	0.057
9	Erythromycin	2 (100%)	0 (0.0%)	3 (100%)	0 (0.0%)	12 (92.3%)	1 (7.7%)	2 (16.7%)	10 (83.3%)	**0.000**
10	Ciprofloxacin	2 (100%)	0 (0.0%)	3 (100%)	0 (0.0%)	13 (100%)	0 (0.0%)	4 (33.3%)	8 (66.7%)	**0.001**
11	Metronidazole	2 (100%)	0 (0.0%)	3 (100%)	0 (0.0%)	13 (100%)	0 (0.0%)	7 (58.3%)	5 (41.7%)	**0.029**
12	Ferrous sulfate	1 (50%)	1 (50%)	3 (100%)	0 (0.0%)	4 (30.8%)	9 (69.2%)	5 (41.7%)	7 (58.3%)	0.186
13	Folic acid	1 (50%)	1 (50%)	3 (100%)	0 (0.0%)	12 (92.3%)	1 (7.7%)	0 (0.0%)	12 (100%)	**0.000**
14	Acetylsalicylic acid	2 (100%)	0 (0.0%)	3 (100%)	0 (0.0%)	11 (84.6%)	2 (15.4%)	0 (0.0%)	12 (100%)	**0.000**
15	Clopidrogrel	1 (50%)	1 (50%)	3 (100%)	0 (0.0%)	9 (69.2%)	4 (30.8%)	0 (0.0%)	12 (100%)	**0.001**
16	Glyceryl trinitrate	0 (0.0%)	2 (100%)	0 (0.0%)	3 (100%)	6 (46.2%)	7 (53.8%)	0 (0.0%)	12 (100%)	**0.020**
17	Propanolol	1 (50%)	1 (50%)	0 (0.0%)	3 (100%)	9 (69.2%)	4 (30.8%)	0 (0.0%)	12 (100%)	**0.002**
18	Hydrochlorothiazide	0 (0.0%)	2 (100%)	1 (33.3%)	2 (66.7%)	6 (46.2%)	7 (53.8%)	0 (0.0%)	12 (100%)	**0.042**
19	Nifedipine	0 (0.0%)	2 (100%)	3 (100%)	0 (0.0%)	11 (84.6%)	2 (15.4%)	0 (0.0%)	12 (100%)	**0.000**
20	Furosemide	1 (50%)	1 (50%)	3 (100%)	0 (0.0%)	13 (100%)	0 (0.0%)	4 (33.3%)	8 (66.7%)	**0.002**
21	Ranitidine	2 (100%)	0 (0.0%)	3 (100%)	0 (0.0%)	13 (100%)	0 (0.0%)	4 (33.3%)	8 (66.7%)	**0.001**
22	Oral dehydration salts	2 (100%)	0 (0.0%)	1 (33.3%)	2 (66.7%)	10 (76.9%)	3 (23.1%)	5 (41.7%)	7 (58.3%)	0.141
23	Glibenclamide	2 (100%)	0 (0.0%)	3 (100%)	0 (0.0%)	13 (100%)	0 (0.0%)	4 (33.3%)	8 (66.7%)	**0.001**
24	Metformin	2 (100%)	0 (0.0%)	2 (66.7%)	1 (33.3%)	11 (84.6%)	2 (15.4%)	3 (25%)	9 (75%)	**0.013**
25	Chlorpromazine HCl	1 (50%)	1 (50%)	1 (33.3%)	2 (66.7%)	11 (84.6%)	2 (15.4%)	0 (0.0%)	12 (100%)	**0.000**
26	Salbutamol	0 (0.0%)	2 (100%)	3 (100%)	0 (0.0%)	13 (100%)	0 (0.0%)	0 (0.0%)	12 (100%)	**0.000**
27	Digoxin	1 (50%)	1 (50%)	3 (100%)	0 (0.0%)	11 (84.6%)	2 (15.4%)	0 (0.0%)	12 (100%)	**0.000**
28	Atorvastatin	0 (0.0%)	2 (100%)	3 (100%)	0 (0.0%)	12 (92.3%)	1 (7.7%)	2 (16.7%)	10 (83.3%)	**0.000**
29	Simvastatin	0 (0.0%)	2 (100%)	0 (0.0%)	3 (100%)	10 (76.9%)	3 (23.1%)	2 (16.7%)	10 (83.3%)	**0.004**
30	Methyldopa	0 (0.0%)	2 (100%)	3 (100%)	0 (0.0%)	13 (100%)	0 (0.0%)	0 (0.0%)	12 (100%)	**0.000**
	**Total**	**32** **(53.3%)**	**28** **(46.7%)**	**66** **(73.3%)**	**24** **(26.7%)**	**311** **(79.7%)**	**79** **(20.3%)**	**68** **(18.9%)**	**292** **(81.1%)**	

*p*-values in bold mean ‘significant’.

## Data Availability

The data presented in this study are available on request from the corresponding author.
